# Abdominal Compartment Syndrome Secondary to Chronic Constipation

**DOI:** 10.1155/2011/562730

**Published:** 2011-08-23

**Authors:** Helene Flageole, Jodie Ouahed, J. Mark Walton, Yasmin Yousef

**Affiliations:** ^1^Department of Pediatric Surgery, McMaster Children's Hospital, Hamilton, ON, Canada L8N 3Z5; ^2^Department of Pediatrics, McMaster Children's Hospital, Hamilton, ON, Canada L8N 3Z5

## Abstract

Abdominal compartment syndrome (ACS) is defined as an elevated intraabdominal pressure with evidence of organ dysfunction. The majority of published reports of ACS are in neonates with abdominal wall defects and in adults following trauma or burns, but it is poorly described in children. We describe the unusual presentation of an 11-year-old boy with a long history of chronic constipation who developed acute ACS requiring resuscitative measures and emergent disimpaction. He presented with a 2-week history of increasing abdominal pain, nausea, diminished appetite and longstanding encopresis. On exam, he was emaciated with a massively distended abdomen with a palpable fecaloma. Abdominal XR confirmed these findings. Within 24 hours of presentation, he became tachycardic and oliguric with orthostatic hypotension. Following two enemas, he acutely deteriorated with severe hypotension, marked tachycardia, acute respiratory distress, and a declining mental status. Endotracheal intubation, fluid boluses, and vasopressors were commenced, followed by emergent surgical fecal disimpaction. This resulted in rapid improvement in vital signs. He has been thoroughly investigated and no other condition apart from functional constipation has been identified. Although ACS secondary to constipation is extremely unusual, this case illustrates the need to actively treat constipation and what can happen if it is not.

## 1. Case Report

An 11-year-old boy presented to the emergency department with a long history of abdominal pain, profound chronic constipation, and encopresis. His new increasing abdominal pain started two weeks prior to presentation with nausea and diminished appetite, preventing him from attending school or tolerating solid foods. 

**Figure 1:**
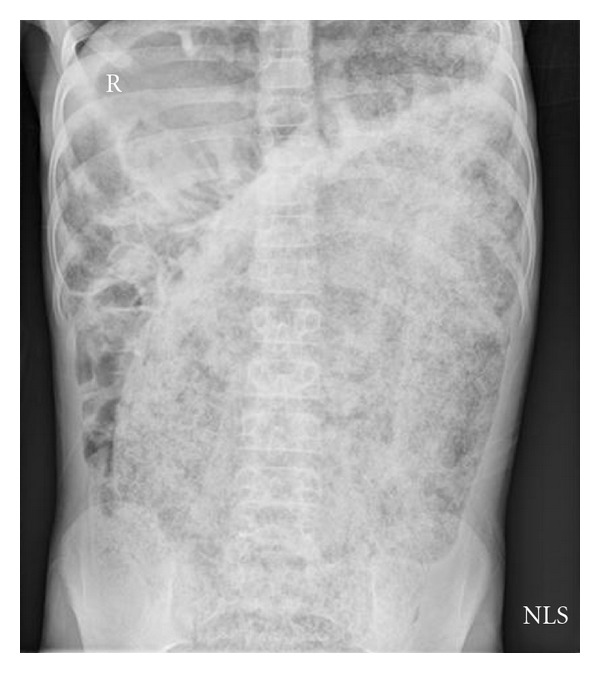
Abdominal X-ray at first presentation confirming profound constipation with large fecaloma.

**Figure 2:**
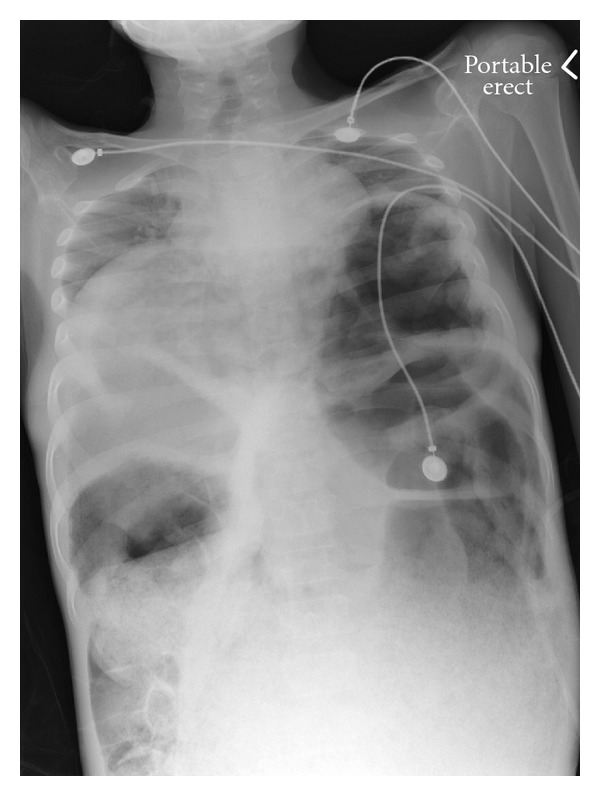
Chest X-ray performed just prior to intubation. Marked elevation of the heart and diaphragm with decreased lung volumes and compressed trachea.

On exam, he was tachycardic but otherwise stable. He looked emaciated, with a hugely distended but nontender abdomen filled with palpable stool. He could not stand straight and rectal exam revealed a rectum filled with hard stools and blood at the anal verge. Abdominal XR confirmed these clinical findings (Figure 1). Within 24 hours, he was admitted because of further deterioration. He received 2 500 mL saline enemas and within 10 minutes of the second enema, he suffered an acute decompensation in clinical status. His abdomen became tense and firm. He developed sudden hypotension (Systolic BP 65–85 mmHg; Diastolic BP 28–76 mmHg), escalating tachycardia (146–174 bpm), acute respiratory distress, with tachypnea of 44 breaths per minute, accessory muscle use, and diminished oxygen saturation of 79% on room air. His mental status also declined, manifested by a GCS score of 13. Full resuscitative measures were implemented, including fluid boluses, endotracheal intubation, mechanical ventilation, and vasopressors. Despite these maneuvers, his worrisome status persisted (Table 1). CXR showed severe elevation of both diaphragms and compression of his heart (giving a false appearance of dextrocardia), decreased lung volumes, and a compressed trachea (Figure 2). Abdominal imaging revealed new air fluid levels, but additional radiologic views showed no evidence of free air. He was taken to the operating room immediately for an emergency manual fecal disimpaction. While being positioned, his legs were cold and mottled and priaprism was noted. A large quantity of stool was manually evacuated and came out under significant pressure shooting out 3-4 feet from his anus. This coincided with striking improvements in his vital signs (Table 2). At thirty minutes, there was dramatic improvement of his mean airway pressure and oxygen saturation measurements. With release of the stool that was under pressure, the need for dopamine support decreased and was discontinued prior to completion of the procedure. Improved ventilation and oxygenation followed immediately, as supplemental oxygen was weaned with successful extubation 6 hours later. Immediately following evacuation, his abdomen was less distended, his skin was no longer mottled, or cool, and priaprism resolved. Bloodwork reflected tissue hypoxia, metabolic and respiratory acidosis, with early coagulopathy. X-rays done postoperatively in the Pediatric Critical Care Unit showed no free air. A peripherally inserted central catheter was placed to facilitate intravenous nutrition support. 

The day following his disimpaction (36 hours later) he suddenly developed free intraabdominal air and was taken to the operating room where a pinhole-sized perforation was identified at the apex of his enormously dilated rectosigmoid. A Hartmann procedure was performed. A descending colostomy was created and biopsies of his bowel were taken. All investigations for Hirschsprung's disease or other intestinal motility disorders were negative. Echocardiogram done following his recovery showed no structural abnormality to the heart. The patient was discharged after 19 days. His colostomy was reversed after seven months, and he continues to do well to date taking daily oral PEG 3350 to maintain regular bowel movements.

## 2. Discussion

Abdominal compartment syndrome (ACS) is defined as an elevated intraabdominal pressure (IAP) with cardiovascular, renal, and pulmonary dysfunction, where abdominal decompression has beneficial effects [1–4]. Above an IAP of 35 mmHg, adults suffer consequences of ACS, but children are thought to have a lower threshold for IAP [3, 5, 6]. Elevated IAP has the potential to progress to ACS with multiorgan dysfunction, and these patients should be closely monitored for deterioration [7, 8]. If ACS develops, medical strategies to reduce IAP can be attempted, but refractory cases require surgical decompression [5]. When there is no time to measure IAP, clinical signs including tensely distended abdomen, impalpable femoral pulses, cyanosis of low extremities, and progressive oliguria and hypoxia are sufficient to justify decompression [4]. If untreated, ACS is associated with 80–100% mortality. With early decompression, mortality rates are still high at 40–60% [4, 9]. 

ACS causes organ failure through direct mechanical effects. Respiratory derangements result when the elevated diaphragm decreases functional residual capacity and increases airway pressures, as depicted in our patient during his acute deterioration [8]. Cardiovascular compromise results from decreased venous return from compression of the heart and the inferior vena cava. Such derangement was first clinically appreciated by our patient's hypotension and tachycardia, then radiologically by his elevated diaphragm and compressed heart. It progressed to cause decreased limb perfusion with reduced venous return and venous hypertension, manifesting as priaprism. The consequences of his increased IAP are well illustrated by his dopamine requirement that immediately resolved following disimpaction. In ACS, renal vein compression impairs kidney function [4, 8]. Oliguria with rising creatinine (40 to 70 Umol/L within 25 hours of presentation) also suggest renal dysfunction. Lactic acidosis and elevated INR reflect endured tissue hypoxia. Our patient's ACS likely resulted from acute and chronic changes when his delicate baseline of a massively distended colon was challenged by the volume of two enemas, resulting in a state of acute decompensation. In the first 24 hours after disimpaction, there was dramatic improvement and no clinical evidence of perforation by the enemas or disimpaction. Most likely this was as a result of a reperfusion injury to the rectosigmoid junction following recovery from the ACS leading to perforation [10]. 

ACS is poorly described in children, and its development from constipation is very seldom reported in the pediatric literature, with only 2 cases published to date. Birkhahn and Gaeta, describe a boy with congenital megacolon who developed ACS from massive colonic dilatation, where surgical decompression resulted in reperfusion injury, coagulopathy, and the patient's fatal demise [7]. Gorecki et al. provide images of a 13-year-old boy who developed ACS from intractable constipation, but do not discuss details of this case [11]. The underlying severe constipation leading to ACS in our patient warrants attention. His profound constipation was resistant to numerous stool softeners and enemas, requiring regular manual disimpaction and frequent nasogastric electrolyte solution. It was so severe that it limited school attendance. More striking is the extent of chronic malnutrition our patient consequently suffered, marked by a low albumin (19 g/L), anemia requiring multiple transfusions, and his development of refeeding syndrome following recovery from disimpaction.

Thorough investigations were undertaken in attempt to identify a primary etiology. Colonic and rectal biopsies were negative for Hirshsprung's disease. Repeat thyroid and celiac screens were negative, and an MRI of his spine ruled out the possibility of a tethered cord. A Shapes study was consistent with a diffuse colonic dysmotility, an intestinal motility study was unremarkable, and anorectal manometry revealed no evidence of outlet dysfunction [12]. Despite extensive testing, a diagnosis of functional constipation still holds. Furthermore, with close monitoring, the treating team has been successful in reinstituting a healthy bowel regimen following his reanastomosis. 

We report a unique case of an otherwise healthy 11-year-old boy with severe longstanding functional constipation that evolved in abdominal compartment syndrome. His rapidly progressive presentation was identified early and managed with immediate resuscitation and prompt manual decompression. As depicted in this illustrative case, the importance of vigilant treatment of habitual constipation cannot be overemphasized. This rare case supports the need for aggressive management of constipation, as suboptimal management has the unfortunate but rare potential to progress to life-threatening conditions such as abdominal compartment syndrome.

## Figures and Tables

**Table 1:** Vital signs at the onset of acute deterioration, compared to those values after positive pressure ventilation, saline boluses, and dopamine.

Vital sign and supportive care	Status at onset of acute deterioration	Status after positive pressure ventilation, IV bolus, and vasopressor
Average HR (HR range)	146 (63–174)	150 (135–182)
Average SBP (systolic range)	77 (50–129)	113 (96–121)
Average DBP (diastolic range)	54 (28–101)	84 (54–97)
Respiratory Rate	44	Intubated
SaO_2_	68–79%	81–84%
Medications		60 mL/kg NS bolus
		Dopamine 10 mcg/kg/min

**Table 2:** Vital signs and dopamine requirement at the start of disimpaction and every 15 minutes thereafter.

Parameters and medications	On arrival to OR	15 min in OR	30 min in OR	45 min in OR	60 min in OR	75 min in OR
HR	145	120	115	120	120	110
BP	125/60	110/55	100/55	90–100/50	90/50	95/50
SaO_2_	89%	84%	90%	100%	100%	100%
ET CO_2_	51%	—	—	—	—	32%
Supplemental O_2_	2 L/min	2 L/min	2 L/min	1 L/min	1 Lmin	1 L/min
MAP	51	51	36	32		30
Medications	Rocorunium 30 mcg					
	Dopamine 10 mcg/kg/min	Dopamine 10 mcg/kg/min	Dopamine 8 mcg/kg/min	Dopamine 4 mcg/kg/min	Off dopamine	Off dopamine
	Midazolam 2 mg	Fentanyl 50 mcg				
